# Structure of the Cyclic Nucleotide-Binding Homology Domain of the hERG Channel and Its Insight into Type 2 Long QT Syndrome

**DOI:** 10.1038/srep23712

**Published:** 2016-03-30

**Authors:** Yan Li, Hui Qi Ng, Qingxin Li, CongBao Kang

**Affiliations:** 1 Experimental Therapeutics Centre, Agency for Science, Technology and Research (A*STAR), Singapore, Singapore; 2 Institute of Chemical & Engineering Sciences, Agency for Science, Technology and Research (A*STAR), Singapore, Singapore

## Abstract

The human ether-à-go-go related gene (hERG) channel is crucial for the cardiac action potential by contributing to the fast delayed-rectifier potassium current. Mutations in the hERG channel result in type 2 long QT syndrome (LQT2). The hERG channel contains a cyclic nucleotide-binding homology domain (CNBHD) and this domain is required for the channel gating though molecular interactions with the eag domain. Here we present solution structure of the CNBHD of the hERG channel. The structural study reveals that the CNBHD adopts a similar fold to other KCNH channels. It is self-liganded and it contains a short β-strand that blocks the nucleotide-binding pocket in the β-roll. Folding of LQT2-related mutations in this domain was shown to be affected by point mutation. Mutations in this domain can cause protein aggregation in *E. coli* cells or induce conformational changes. One mutant-R752W showed obvious chemical shift perturbation compared with the wild-type, but it still binds to the eag domain. The helix region from the N-terminal cap domain of the hERG channel showed unspecific interactions with the CNBHD.

The human ether-à-go-go (EAG) related gene (hERG) potassium channel belongs to the KCNH family that also contains EAG and EAG-like (ELK) potassium channel sub-families^1^. The EAG and ELK channels are playing important roles in tumor progression and neuronal excitability^2^,^3^. The hERG channel is crucial for heart function by contributing to the fast delayed-rectifier potassium current (I_kr_) during the repolarization phase of the ventricular action potential^4^,^5^. Inherited or drug induced loss-of-function mutations in hERG channel can result in type 2 long QT syndrome (LQT2), a disease with a risk of cardiac arrhythmias and sudden death^6^,^7^. Mutations in hERG potassium channel contribute to approximately 30% of identified long QT syndrome (LQTS) mutations^8^. Several drugs have been withdrawn from the market due to their inhibitory effect on the hERG channel activity.

KCNH channels are voltage-gated potassium channels whose function is regulated by changes in the membrane potential^9^. The hERG protein contains a voltage-sensor domain that is formed by transmembrane segments S1–S4 and this domain can sense the changes of membrane potential across the cell membrane^10^. It also contains a pore domain that is formed by S5–S6 to form a pore for potassium transport across the cell membrane. The hERG protein also contains a long N-terminus formed an eag domain (a cap domain and a Per-Arnt-Sim (PAS)) and a long linker region between the PAS domain and the first transmembrane segment S1^11^,^12^. There is a C-linker region and a cyclic-nucleotide-binding homology domain (CNBHD) in the C-terminal region of the hERG channel^1^,^13^ (Fig. 1A). CNBHDs of KCNH channels show structural similarity to the cyclic nucleotide-binding domains (CNBDs) of other channels such as cyclic nucleotide-gated (CNG) and hyperpolarization-activated cyclic nucleotide-modulated (HCN) channels. Unlike the HCN channels, KCNH channels do not bind to cAMP because its nucleotide binding pocket is occupied by a short β-strand^1^,^13^,^14^,^15^,^16^,^17^.

Accumulated studies have shown that the domain-domain interactions in hERG channel contribute to its gating property that is important for heart function^18^. Biochemical and biophysical studies have shown that the eag domain interacts with both CNBHD and the linker between transmembrane segments S4 and S5^19^,^20^,^21^. LQT2-related mutations have been identified in both PAS domain and CNBHD. Interrupting the domain-domain interactions may be one of the mechanisms to cause LQT2^15^. For example, R56 in hERG was predicted to be important for interaction with CNBHD based on the crystal structure of mouse mEAG channel and our recent study confirmed that R56Q mutation weakened its interaction with the CNBHD^15^,^16^. Structural studies have provided valuable information to understand channel function. Despite of the importance of hERG channel in heart and drug discovery, only structural information for its N-terminal PAS domain^11^,^12^,^22^,^23^,^24^, the S4–S5 linker^25^,^26^, and the channel region^27^,^28^,^29^ is available so far.

In this study, we present the solution structure of hERG CNBHD. It is shown to adopt a similar fold to other CNBHDs of KCNH channels. It has a short β-strand (β9) that blocks the nucleotide-binding pocket. Effect of disease-related point mutations on the structure of this domain was also investigated. We show that mutations in CNBHD of hERG channel could cause conformational changes that can affect protein stability. We also found that the N-terminal helix of hERG may require the PAS domain to interact with the CNBHD.

## Results

### Structure of the hERG CNBHD

**Figure 1:**
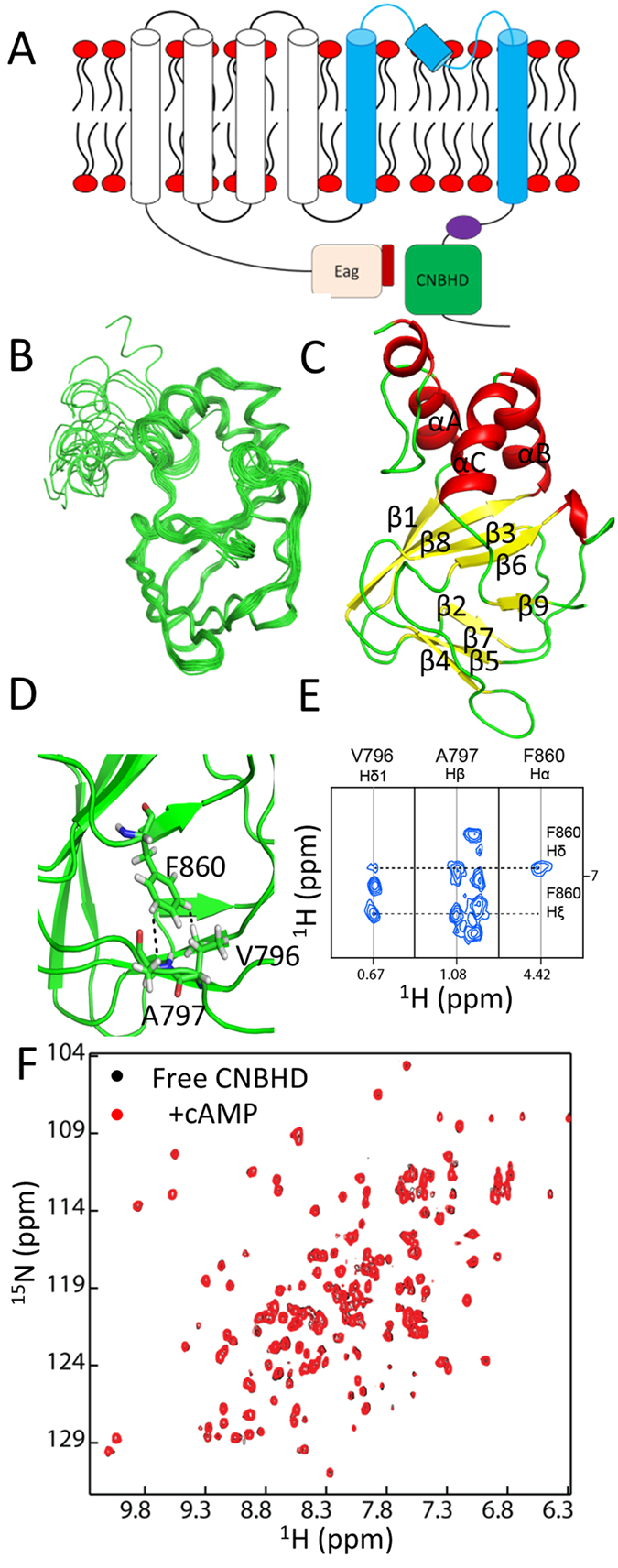
Structure of CNBHD. (**A**), Cartoon of hERG channel. The voltage sensor domain (transmembrane segments S1–S4), pore domain, the PAS domain, the cap domain, and CNBHD domain are labeled in white, blue, gray, red, and green, respectively. (**B**), overlay of 20 lowest energy structures of hERG CNBHD. (**C**), Ribbon representation of one of the conformers of hERG CNBHD. (**D**), F680 interaction network with residues in the β-roll. Residues having NOEs with the side chain of F680 are labeled and the NOEs observed are indicated as dashed lines. (**E**), NOEs observed between F860 and other residues. (**F**). ^1^H-^15^N-HSQC spectra of 0.2 mM ^15^N-labeled hERG CNBHD in the absence (black) and presence of 2 mM cAMP (red). One ^15^N-labled hERG CNBHD was prepared. The stock solution of cAMP was made by dissolving cAMP into water to a 100 mM solution. ^1^H-^15^N-HSQC spectra of CBNHD in the absence and presence of cAMP was obtained and compared. No significant chemical shift perturbation was observed.

One of the challenges to study the structure and function of the CBNHD of the hERG channel *in vitro* is to obtain sufficient protein samples. Using a systematic deletion method, we were able to express and purified a construct containing residues R734 to D864 in *E. coli* for structural studies^16^. This construct contains only the CNBHD and exists as a monomer in solution because of lacking the C-linker region that may be important for protein oligomerization^1^. This construct exhibited a well-resolved ^1^H-^15^N-HSQC spectrum and its backbone assignment was obtained using conventional 3D-experiments and the side chain assignments were obtained for structural studies. The assignment has been deposited in BioMagResBank with accession number 25805. The solution structure of hERG CNBHD was solved using restraints including NOEs derived from a ^15^N- and a ^13^C-edited NOESY experiments, dihedral angles obtained from TALOS+^30^, and hydrogen bond restraints derived from an H-D exchange experiment (Fig. 1B, and Table 1). The ensemble of 20 calculated structures demonstrated a backbone root mean square deviation (RMSD) of 0.44 Å for the backbone atoms and 1.01 Å for the heavy atoms (Fig. 1B,C).

### CNBHD adopts a similar fold to other KCNH channels

The calculated structure reveals that the CNBHD of the hERG channel contains 9 β-strands and 3 helices (Fig. 1B,C). The first 8 β-strands form a β-roll which is a common fold among the canonical cyclic nucleotide binding proteins (Fig. S1). The 3 α helices including αA, αB, and αC are localized at one side of the β-roll (Fig. 1C). The hERG CNBHD contains a short strand-β9 formed by residues F860–R863 (Fig. 1D,E). This short β-strand was shown to sever as a “self-ligand” to block the entry of cAMP into the binding pocket in the crystal structures of KCNH channels^1^,^13^,^15^. When we added cAMP to a ^15^N-labeled CNBHD of the hERG channel, no obvious chemical shift perturbation was observed, confirming that purified hERG CNBHD does not bind to cAMP in solution (Fig. 1F). The short β-strand blocks the entry of cAMP into the binding pocket through the side chain of F860 (Fig. 1D). For the CNBHDs of KCNH channels, the amino acid at this position is either Phe or Tyr (Fig. S2)^1^,^13^,^15^. The side chain of F860 can form a net work of interactions with residues in the β-roll, evidenced by the observed NOEs between its side chain and the nearby residues such as V796 and A797 (Fig. 1D,E). In addition to side chain of Phe 860, other amino acids around the β-roll do not favor cAMP binding. For a canonical cyclic nucleotide-binding protein such as HCN2 channel, the β-roll cavity is the cyclic nucleotide binding site and this β-roll displays a positively charged surface (Fig. S1), which favors the molecular interaction with negatively charged cyclic nucleotides (Fig. S1). For the hERG channel, its CNBHD is negatively charged (Fig. S1), which is similar to other KCNH channels. This negatively charged surface does not favor interactions with negatively charge cyclic nucleotides. The overall folding of the CBNDH is very similar to those of mouse EAG, zebrafish ELK, and mosquito ERG channels (Fig. 1, Fig. S3)^1^,^13^,^15^. Superimposed structures of the CNBHDs of mouse EAG and hERG channels revealed an RMSD of 2.4 Å for the backbone atoms (Fig. S3). Further sequence analysis of the hERG CNBHD shows that it contains a hydrophobic sequence (V_794_VVAIL_799_). Although the function of this region is not known, its hydrophobic nature may make this domain unstable under certain conditions. Interestingly, this region is in the interface of the eag/CNBHD complex based on previous structural study of the mouse eag channel^15^,^16^, suggesting that the eag domain may prevent this region exposed to the solvent.

### Dynamics of the CNBHD

**Figure 2:**
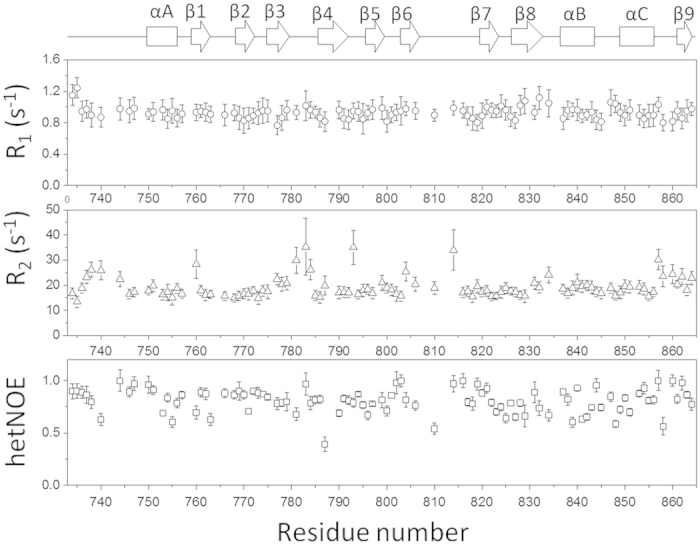
Relaxation analysis of the CNBHD of the hERG channel. R_1_, R_2_ and hetNOE were plotted against residue number. The data were collected on the Bruker 700 MHz magnet equipped with a cryoprobe.

The CNBHD adopts a similar fold to other KCNH channels (Fig. 1, Fig. S3). To understand its dynamics in solution, ^15^N longtitudinal (R_1_), transverse (R_2_), and hetNOE values were obtained. The results are shown in Fig. 2 for the assigned and resolved residues in the ^1^H-^15^N-HSQC spectrum. Overall, the CNBHD of the hERG channel forms a stable structure in solution. The relative lower hetNOE values (less than 0.8) for residues from 820 to 850 suggest that these residues may not be rigid in solution, which may be sensitive to point mutation. Residues between 780 and 785 exhibited higher R_2_ values than the average, suggesting that they may have external exchanges. The average R_1_ and R_2_ values for the CNBHD are 0.92 and 19.3 S^−1^, respectively. The estimated correlation time based on R_2_/R_1_ is approximately 12.2 ns, which is consistent with a monomeric protein with a molecular weight of 17.5 kDa.

### Effect of disease-related mutation on CNBHD folding

**Figure 3:**
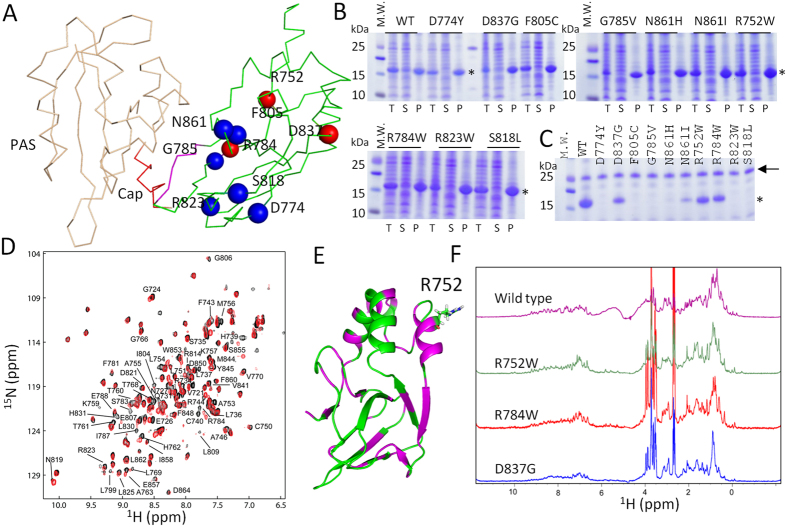
Effect of mutation on CNBHD. (**A**), Structural model of the eag domain and CNBHD complex of the hERG channel. This model was built by aligning the solution structure of CNBHD obtained in this study and the eag domain of hERG (PDB 4HP9) to the eag/CNBHD complex of mouse EAG channel (PDB 4LLO). 10 selected LQT2 mutations are shown in sphere. Mutants that can be purified from *E. coli* are labeled in red. The six hydrophobic residues in the β5 are labeled in magenta. (**B**), SDS-PAGE analysis of the solubility of CNBHD wild type and mutations expressed in *E. coli*. M.W. is the molecular weight standard. T is total cell lysate. P and S are the pellet and supernatant after centrifugation, respectively. (**C**), Purification of CNBHD and its mutants. The band corresponding to CNBHD is labeled with an asterisk. Arrow indicates a bacterial protein with a molecular weight of 25 kDa. (**D**). Overlay of the ^1^H-^15^N-HSQC spectra of the CNBHD (black) and the R752W mutant (red). The residues showed chemical shift changes are labeled with residue name and sequence number. (**D**). Affected residues are mapped onto the structure of the CBNHD. The residues with chemical shift perturbation are shown in magenta. Residue R752 is shown in sticks and labeled. (**E**). ^1^H-NMR spectra of the CNBHD wild type and its three mutants.

Mutations in hERG channel can cause LQT2 and several disease-related mutations have been identified in the CNBHD. Breaking the molecular interaction between the eag domain and the CNBHD was considered as one of the mechanisms to cause LQT2 because several mutations are localized at the binding interface of eag domain and CNBHD^15^. A recent study reported a comprehensive analysis of LQT2-linked mutations^31^. It was proposed that most mutations in the CNBHD can cause channel mis-trafficking^31^. To understand the mechanism of LQT2-related mutations in CNBHD, we attempted to test the folding of the mutants in *E. coli*. We over-expressed 10 LQT2-related mutations in *E. coli*. These chosen mutants are spread all over the CNBHD domain (Fig. 3A). Our results show that all these mutants can be expressed in *E. coli* (Fig. 3B). Compared with the wild type, the solubility of the mutants were reduced dramatically, suggesting that single mutation in CNBHD destabilized its structure to cause protein aggregation or misfolding (Fig. 3B). Six out of the 10 mutants were demonstrated to be insoluble when they were expressed in *E. coli* (Fig. 3B,C). These mutants were shown to be traffic-deficient^31^, suggesting that folding of CNBHD is important for channel trafficking. Four mutants including N681I, D837G, R752W, and R823W can be purified from *E. coli* (Fig. 3C). The yield of N681I is too low to be used for further characterization. N681 is located at the binding interface with the eag domain. Low solubility of N681I or N681H may affect the interaction with eag domain. Interestingly, the other three mutants are not localized at the interface of eag domain-CNBHD complex, indicating that LQT2 arisen from these mutations is caused by incorrect folding rather than perturbation of its interaction with the eag domain.

### Point mutation in CNBHD affects its folding

**Figure 4:**
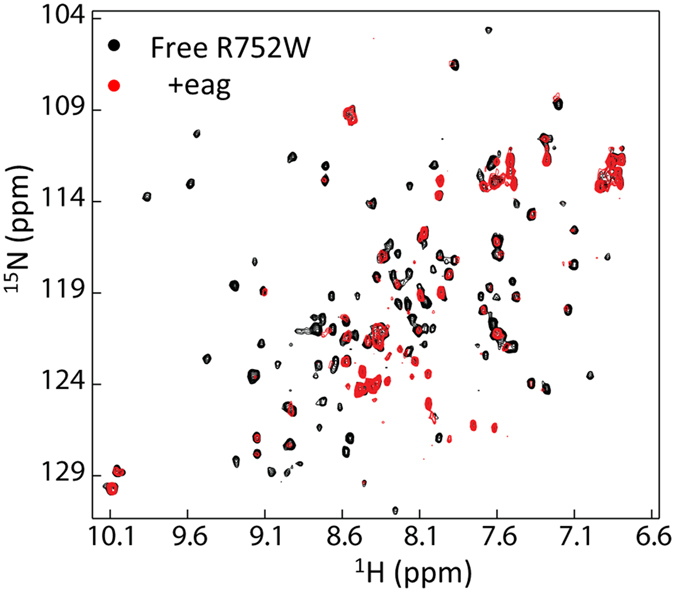
Interaction between the eag domain and the CNBHD. ^1^H-^15^N-HSQC spectra of R752W mutant in the absence and presence of 2 times of un-labeled eag domain.

To understand the folding of the three purified mutants, we first collected their ^1^H-^15^N-HSQC spectra. Only the R752W exhibited good quality spectrum (Fig. 3D). The spectral quality for the other two mutants was poor due to the low sample concentration. Overall, the R752W mutant exhibited well dispersed cross peaks in the spectrum, suggesting its folding in solution. We then compared the ^1^H-^15^N-HSQC spectra between wild type and the R752 mutant (Fig. 3D). Quite a few residues exhibited chemical shift perturbation and line broadening, which indicates that this point mutation affected their structure or their local environment. Based on the assignment of the wild type protein, residues caused by mutation are mapped to the structure of CNBHD (Fig. 3E). In addition to the residues close to R752, several residues that are far away are affected by the point mutation. R752 is a positively charged residue with its side chain exposed to the solvent (Fig. 3E). Mutation from Arg to Trp that contains a hydrophobic side chain may not favor the folding of the CNBHD, which can affect the chemical environmental of other residues. We then tested whether this mutant still interacts with the eag domain. Surprisingly, R752W mutant still showed interactions with the eag domain (Fig. 4). Compared with the CBNHD wild type, more residues from the eag were affected then it binds to R752W. These results suggest that the folding of the CNBHD of the hERG channel is critical for the channel function. Mutation in the CNBHD can cause protein aggregation or conformational to affect its interaction with the eag domain. Strengthen or weaken the interaction will affect channel gating or trafficking. This protein-protein interaction may be critical for channel trafficking.

^1^H NMR has been used for testing the folding of a purified protein in solution^32^. Although the peaks in the ^1^H spectrum are difficult to assign due to the signal overlap and the complexity of protein proton signals, ^1^H-based NMR spectrum is still useful to understand structural and conformational changes of a protein induced by a mutation or a ligand interaction. We collected and compared ^1^H NMR spectra of the three mutants and the CNBHD wild type (Fig. 3F). All these mutants exhibited dispersed proton signals in both amide and aliphatic regions of the spectra, suggesting their folding in solution (Fig. 3F). Surprisingly, obvious chemical shift difference between wild type and mutants was observed. We focus on the methyl proton region that contains signals from the protons attached to the carbon side chain of Leu or Val. In the wild-type spectrum, there are several methyl proton signals observed (Fig. 3F). Fewer signals were observed or chemical shift perturbations were observed for the mutants (Fig. 3F). This result suggests that there may be conformational changes caused by the point mutation. The 1D and 2D NMR results suggest that the CNBHD of hERG channel is very sensitive to the point mutations, which can induce conformational changes to affect channel trafficking.

### The helix from the N-terminal cap domain shows unspecific binding to CNBHD

**Figure 5:**
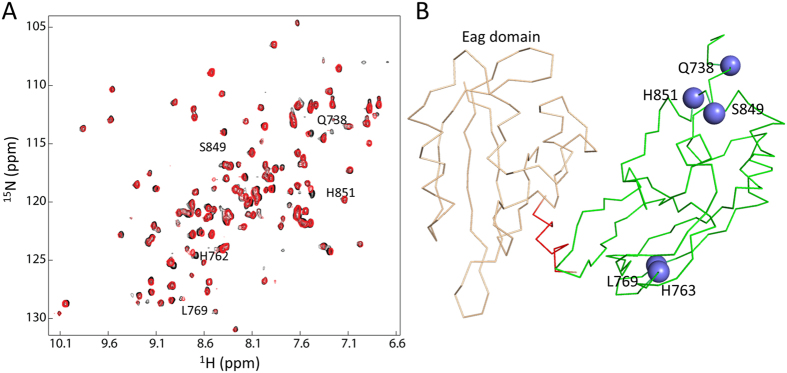
Interaction between the cap helix and the CBNHD. (A). Overlay of the ^1^H-^15^N-HSQC spectra of the CNBHD in the absence (black) and presence of 0.5 mM peptide (red). Residues showed chemical shift changes are labeled with residue name and sequence number. (B). Affected residues of the CNBHD by addition of the peptide. The residues showed chemical shift perturbations are mapped onto the structure of the CNBHD and are shown in blue spheres.

The helix in the cap domain of hERG channel was shown to be involved in the molecular interaction with the CNBHD^15^,^16^. The cap domain was identified by recent structural studies using solution NMR spectroscopy^22^,^24^,^25^. It was shown to be rigid in solution and interact with both PAS domain and the CNBHD^16^,^33^. Residues including D16, R20, and F22 from the cap domain are shown to be involved in binding with the CNBHD^16^. To further confirm its interaction with the CBNHD, titration of a peptide derived from the cap domain helix (T13-E23) to a ^15^N-labeled CNBHD was carried out (Fig. 5A). Although chemical shift perturbation of the CNBHD was observed when the peptide was present, no binding interface on the CNBHD was identified, indicating that the binding is not specific (Fig. 5B). The unspecific interaction between the cap helix peptide and CNBHD suggested that the PAS domain or the N-terminal residues preceding the cap helix may be critical for the function of the cap helix, which is in agreement with the study showing that mutations in the first 11 residues such as R7A shifted the voltage dependence dramatically to more depolarized potentials^15^.

## Discussion

In this study, we showed that the structure of the CNBHD of the hERG channel does not favor the interactions with cyclic nucleotide (Fig. 1), which is also confirmed by the NMR experiment (Fig. 1). Like other KCNH channels, the CNBHD contains a short β-strand (β9) in which F860 acts as an “intrinsic ligand” to block the entry of the cyclic nucleotides into the pocket (Fig. 1). Based on current study and previous X-ray structural studies^1^,^13^,^15^, it is evident that the CNBHD of the KCNH channels contains this unique β-strand, which makes this family of channels independent of the cyclic nucleotides^1^,^14^,^15^. Despite the structural similarities between the CNBHD of the hERG channel and other KCNH channels, the RMSD between hERG and mouse CNBHDs was 2.4 Å for the backbone atoms (Fig. 1). The orientations of the three helices are slightly different in the structures (Fig. S3). The explanation for such difference may arise from the fact that the construct used in current study does not contain the C-linker region that may affect the orientation of the helices slightly. Nevertheless, the current structure still explains the function of the CNBHD of the hERG channel.

**Figure 6:**
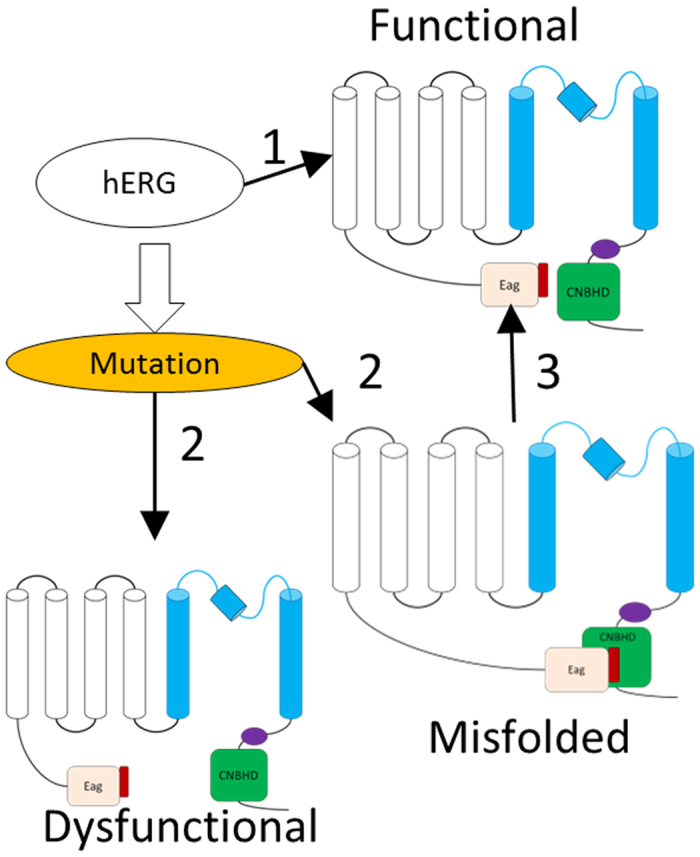
Model of the role of eag domain and the CNBHD domain in hERG channel. Wild type channel is functional (top panel). (**1**) indicates that the channel is functional through the eag domain and the CNBHD interactions. (**2**) means that mutation in the CNBHD can cause channel mis-folding or dysfunctional. The dysfunctional channels may not be rescued due to lose of domain-domain interactions. (**3**) means that misfiled channel can be rescued under certain conditions because the eag domain and the CNBDH still have interactions.

The model of CNBHD/eag complex of the hERG channel can be obtained based on the X-ray structure of the complex of the mouse EAG channel^16^ (Fig. 3). We could not obtain the binding affinity between the eag domain and the CNBHD using other biophysical methods such as isothermal titration calorimetry. Our previous NMR study also confirmed the binding interface and the interaction was undergoing intermediate exchange, which suggests that the affinity is in μM to mM range^16^. Perturbation of the interaction between the eag domain and the CNBHD may be one of the reasons to cause LQT2^15^. R56Q-a LQT2 mutation in the eag domain showed reduced interaction with the CNBHD, supporting that this mutation can affect eag-CNBHD interaction^16^. When we expressed ten LQT2-related mutations in the CNBHD in *E. coli*, most of them were not soluble (Fig. 3). The mutants that can be purified from *E. coli* showed structural changes (Fig. 3). The R752 showed interaction with the eag domain with more residues affected on the eag domain (Fig. 4), suggesting that the binding interface may be slightly changed due to the mutation. All these results imply that the folding of the CNBHD is critical for channel function such as trafficking. The β-5 of the CNBHD contains a hydrophobic sequence formed by residues V794 to L799 (VVVAIL) (Fig. 1). This sequence is localized at the interface of the eag domain/CNBHD complex. The interaction between these two domains may prevent exposure of this hydrophobic region to the solvent, which may be essential for channel trafficking and maturation. Mutation in the PAS domain or the CNBHD may affect their interactions, which makes this hydrophobic region exposed to the solvent to affect the channel function. Therefore, the stability of the CNBHD and its interaction with the eag domain may be crucial for the channel function. Under normal conditions, the eag domain interacts with the CNBHD and the channel is functional (Fig. 6). Mutation in the CNBHD can result in CNBHD aggregation or misfolding in solution. The misfolded mutant will lose interactions with the eag domain, which makes channel dysfunctional. A mutation in the CNBHD can also cause some conformational changes such as R752W, which affected its interaction with the eag domain to make the channel misfolded. The misfolded channel can be rescued under certain conditions (Fig. 6). Our study on the disease-related mutants suggested that these mutants that can be purified from *E. coli* can be rescued under some conditions, while the ones that are insoluble in *E. coli* might be difficult to be rescued. Further study on the affinity between the eag domain and the CNBHD mutants using other biophysical methods will be useful to understand their interactions. Protein posttranslational modification is critical for protein function and stability. Mature hERG channel is observed to be glycosylated^34^. It will also be useful to investigate the folding of LQT2-related mutants that are in glycosylated form because all the CNBHD mutants used in this study are not posttranslational modified. It has been noted that there are more than 10 LQT2-related mutations in CNBHD. One of the mutants R744fs has been shown to fail in channel assemble, but it can be glycosylated under certain conditions^35^, suggesting the protein-protein interaction model proposed in Fig. 6 may not be able to explain all the LQT2-related mutations. Large scale structural investigation of the disease-related mutation will provide more insight into LQT2, which will also be useful for designing a strategy to rescue LQT2 patients.

In summary, we present the structure and dynamic study of the CNBHD of the hERG channel. This domain has a similar fold to other KCNH channels. The presence of β9 and its negatively charged β-roll cavity prevent its interaction with cyclic nucleotides. Several LQT2-related mutations in CBNHD were shown to be insoluble in *E. coli* or have conformational changes, suggesting that the folding of this domain is critical for the channel function.

## Experimental Procedures

### Protein expression and purification

The cDNA encoding the hERG potassium channel was synthesized (Genscript). Several constructs containing CNBHD were made and only residues R734 to D864 (referred as CNBHD) was able to be expressed and purified in *E. coli*. The cDNA encoding this region was cloned into PNIC28-Bsa4^36^ and pET29b, respectively. The resulting plasmids produce a recombinant protein that contained an N-terminal histidine tag, a TEV cleave site and CNBHD. The plasmid for expressing CNBHD was transformed into BL21(DE3) Rosetta T1R or BL21(DE3) cells. Protein was induced and purified as previously described^22^. Briefly, 20 μl of glycerol stock of *E. coli* cells was inoculated in 50 ml of M9 medium supplied with 30 μg/ml kanamycin. The overnight culture was transferred into 1 L of M9 medium. Induction was performed by adding IPTG to 1 mM final concentration with additional shaking at 200 rpm and 18 °C overnight when the optical density (OD_600_) reached 0.6–0.8. *E. coli* cells were harvested by centrifugation at 10,000 × g and 4 °C for 10 min. The cell pellet was suspended in a lysis buffer containing 20 mM sodium phosphate, pH 7.8, 300 mM NaCl and 2 mM β-mercaptoethanol. Cells were broken by sonication in an ice bath and the cell lysate was cleared by centrifugation at 40,000 × g and 4 °C for 20 min. The supernatant was mixed with Ni^2+^-NTA resin (Qiagen) and purified using a gravity column. Protein was eluted with an elution buffer that contained 300 mM imidazole, pH 6.5, 500 mM NaCl and 2 mM β-mercaptoethanol. Protein was further purified using a gel filtration chromatography in a buffer that contained 20 mM sodium phosphate, pH 7.2, 150 mM NaCl, and 1 mM DTT. Protein was concentrated to 0.5–0.8 mM. The N-terminal tag was not removed in the study because the CNBHD was not stable when the fusion tag was removed by protease digestion. The eag domain of hERG was expressed and purified as previously described^33^,^22^. LQT2-related mutations were made by site-directed mutagenesis. The mutants were expressed and purified in *E. coli* using the same method as wile type. Same amount of induced cells were used for testing solubility. During protein purification, same amount of elution buffer was used to elute mutants from resin and SDS-PAGE analysis was conducted to test whether protein can be purified.

### NMR experiments

Backbone chemical shift assignment was conducted using the experiments described previously^16^. Side chain resonance assignment was conducted using three dimensional (3D) experiments including HBHACONH, HCCONH, CCONH, HCCH-TOCSY experiments. ^15^N- and ^13^C-edited NOESY experiments were collected for the NOE restraints. Hydrogen-deuterium (H-D) exchange experiment was carried out to obtain hydrogen bond restraints. Briefly, a ^15^N-labeled protein sample was prepared in the sample buffer and frozen in liquid nitrogen immediately after purification. The sample was then lyophilized at a low temperature and low pressure. D_2_O (99.9%) was then added to the lyophilized sample and ^1^H-^15^N-HSQC spectra were acquired. The cross peaks in the ^1^H-^15^N-HSQC spectrum are resides protected from exchanges. Hydrogen bond restraints were set up based on the NOE assignment and the H-D exchange experiment. All the experiments were collected on a Bruker 600 MHz or 700 MHz magnet equipped with a cryogenic probe. The spectra were acquired using Topspin (2.1) and processed with NMRPipe^37^, Topspin and visualized using NMRView^38^ and CARA (http://www.cara.nmr-software.org/downloads/). For peptide and CNBHD binding study, the 11-residue peptide (TFLDTIIRKFE) corresponding to the N-terminal helix was synthesized. ^1^H-^15^N-HSQC spectra of 0.2 mM ^15^N-1abeled CNBHD in the absence and presence of 0.5 mM peptide were recorded and compared.

### Relaxation measurement

The herteronuclear NOE (hetNOE), ^15^N longitudinal R_1_, and transverse R_2_ relaxation rates experiments^39^ were carried out at 298 K using a ^15^N-labeled sample in the NMR buffer on a Bruker Avance II 700 MHz spectrometer equipped with a cryoprobe. For R_1_ measurements, the relaxation delays of 50, 100, 200, 400, 600, 800, 1000, 1200, 1400, and 1600 ms were recorded as performed previously. For R_2_ measurements, the data were acquired with delays of 16.9, 34, 51, 68, 85, 102, 119, 136 and 153 ms. The hetNOE data were obtained using two datasets with and without initial proton saturation for a period of 3 s. The spectra were processed and analyzed as previously described^33^.

### Structure determination

The dihedral angle restrains were predicted using TALOS+ based on the chemical shifts^30^. The NOE restraints were obtained from NOESY experiments. Peaks from a 3D ^1^H-^15^N-NOESY and a 3D-^1^H-^13^C-NOESY experiments were picked and assigned manually. Peak intensity was converted to distance restrains using CYANA3.97 (obtained from Prof Peter Guntert) and CYANA2.1^40^. Hydrogen bond restraints were derived from H-D exchange experiment. The upper and lower distances used in hydrogen bond restraints were set to 2.8 and 1.8 Å, respectively. Structure determination was carried out using CYANA3.97 using the collected restraints. Standard simulated annealing was scheduled with 10,000 torsion angle dynamics steps. One hundred structures were calculated and 20 structures with lowest final target function values were obtained. Protein structure was analyzed using PROCHECK-NMR^41^ and visualized using MOLMOL^42^ and PyMOL (www.pymol.org).

## Additional Information

**How to cite this article**: Li, Y. *et al.* Structure of the Cyclic Nucleotide-Binding Homology Domain of the hERG Channel and Its Insight into Type 2 Long QT Syndrome. *Sci. Rep.* **6**, 23712; doi: 10.1038/srep23712 (2016).

## Supplementary Material

Supplementary Information

## Figures and Tables

**Table 1:** Summary of the 20 structures of CNBHD of hERG channel.

	
Number of unambiguous NOEs	3236
Short range (|i−j|≤1)	2421
Medium-range (1<|i−j|<5)	231
Long-range (|*i−j|*>4)	584
Number of dihedral angle constraints	232
Number of hydrogen-bond restraints	62
Number of restraint violations^a^	
Total number of restraint violations >0.5 Å	0
Total number of dihedral angle constraints >5°	0
Ramachandran plot statistics^b^ (%)	
Residues in most favored regions	76.1
Residues in additionally allowed regions	22.1
Residues in generously allowed regions	1.8
Residues in disallowed regions	0
Average RMSD to mean (Å)	
Backbone (residues 765–864)	0.44 ± 0.16 Å
Heavy atoms (residues 765–864)	1.01 ± 0.22 Å

^a^There are no distance violations greater than 0.5 Å or dihedral angle violations greater than 5°.

^b^The Ramachandran plot was obtained using PROCHECK-NMR based on the conformer with lowest energy. The analysis was conducted for residues R734 to D864.
