# Supplementary material for: Structure of the Cyclic Nucleotide-Binding Homology Domain of the hERG Channel and Its Insight into Type 2 Long QT Syndrome
Source: Sci Rep. 2016 Mar 30;6:23712. doi: 10.1038/srep23712 (PMC4812329; doi:10.1038/srep23712)
Supplement: Supplementary Information [file srep23712-s1.pdf]

Supplementary Information

Structure of the Cyclic Nucleotide-Binding Homology Domain of the hERG Channel and Its Insight into Type 2 Long QT Syndrome

Yan Li[1], Hui Qi Ng[1], Qingxin Li[2*], and CongBao Kang[1,*]

[1]Experimental Therapeutics Centre, Agency for Science, [2]Institute of Chemical & Engineering Sciences, Agency for Science, Technology and Research (A*STAR), Singapore, Singapore

To whom correspondence should be addressed: CongBao Kang, 31 Biopolis Way Nanos, #03-01, Singapore. Tel: 65-64070602; Fax: 65-64788768; Email: cbkang@etc.a-star.edu.sg; Qingxin Li, email: li_qingxin@ices.a-star.edu.sg

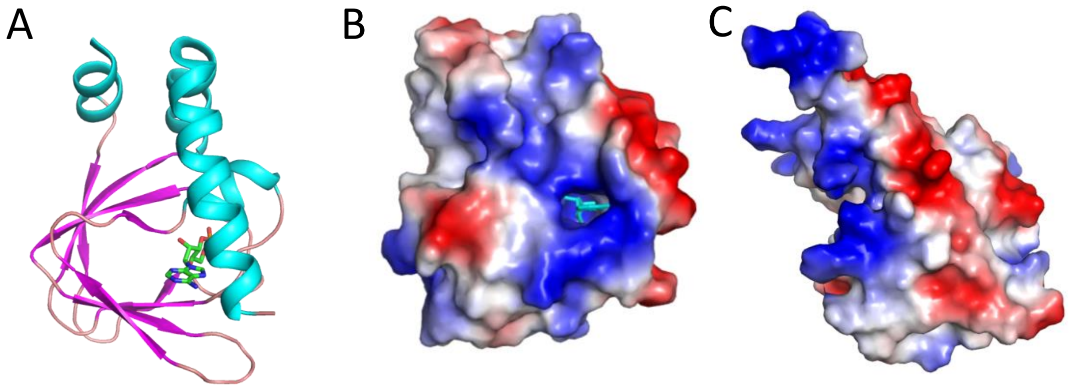

Figure S1 Structure basis for cAMP binding. A, Structure of mouse HCN2-cAMP complex structure (PDB 1Q5O). cAMP is shown in sticks and green. B, C, Electrostatic potential surface of CNBHDs of mouse HCN2 channel (PDB 1Q5O), and hERG CNBHD in the same orientation.

```
zebrafish ELK   NKELLQLPLFESASRGCLRSLSLIIKTSFCAPGEFLIRQGDALQAIYFVCSGSMEVLKDN  671
mouse EAG1      RKVFKEHPAFRLASDGCLRALAMEFQTVHCAPGDLIYHAGESVDSLCFVVSGSLEVIQDD  632
human ERG       RSLLQHCKPFRGATKGCLRALAMKFKTTHAPPGDTLVHAGDLLTALYFISRGSIEILRGD  793
Mosquito ERG    RNLLNNCSAFEAASPGCLRALSLKFKTTHAPPGDILVHKGDVLTYLYFIARGSIEILKDD  660
                 . : .   *. *: ****:*:: ::* .. **: : : *: :  : *:  **:*::: :

Zebrafish ELK   TVLAILGKGDLIGSDSLTKEQVIKTNANVKALTYCDLQYISLKGLREVLRLYPEYAQKFV  731
mouse EAG1      EVVAILGKGDVFGDVFWKEATLAQSCANVRALTYCDLHVIKRDALQKVLEFYTAFSHSFS  692
human ERG       VVVAILGKNDIFGEPLNLYARPGKSNGDVRALTYCDLHKIHRDDLLEVLDMYPEFSDHFW  853
mosquito ERG    VVMAILGKDDIFGENPCIHSTLGKSNSNVKALTYCDLHKIHRDDLLDVLDLFPEFYDSFV  720
                 *:***** *::*.         ::  .:*:*******: *  . * .** ::  : . *

zebrafish ELK   SEIQHDLTYNLRE  744
mouse EAG1      RNL--ILTYNLRK  703
human ERG       SSL--EITFNLRD  864
Mosquito ERG    NSL--EITYNMRD  731
                 .:   :*:*:*.
```

Figure S2 Sequence alignment of CNBHDs of several KCNH channels whose structures have been solved. The conserved residue is highlighted in green. Multiple sequence alignment was carried out using clustal Omega (http://www.ebi.ac.uk/Tools/msa/clustalo/).

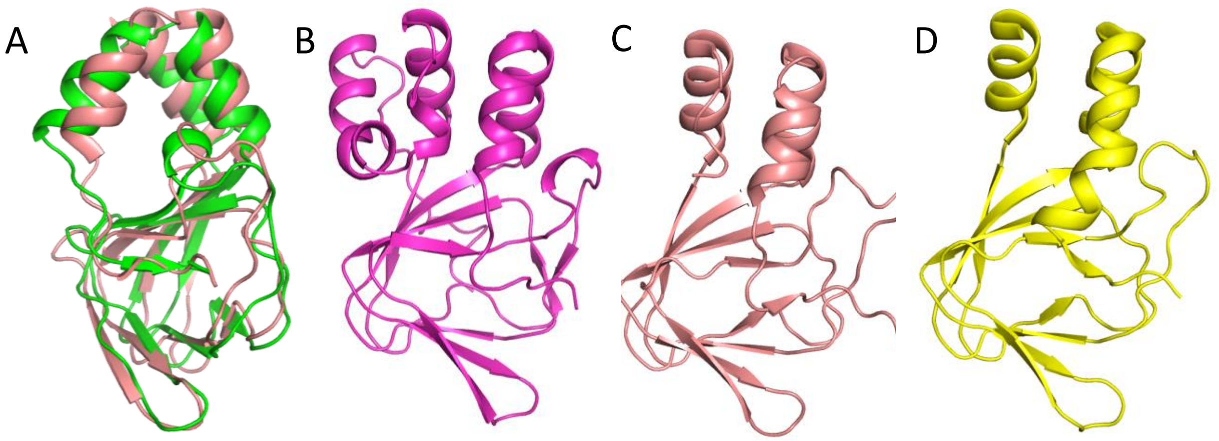

Figure S3 Structural comparison of hERG CNBHD with other ion channels. A, Overlay of the CNBHDs of hERG channel and the mouse channel. The CNBHD of the mouse channel (PDB id 4LLO) is shown in pink. The CNBHD of the hERG channel is shown in green. B, structure of CNBHD of mouse EAG1 (PDB 4LLO). C, structure of a mosquito ERG channel (PDB 4L11). D, structure of zebrafish ELK channel (PDB 3UKN). For clarity, only partial structures of the CNBHDs from the x-ray structures are shown. All the figures were made using PyMOL (www.pymol.org).
